# Eye Disease Prevalence and VF-14 Validation Among Patients Experiencing Homelessness and Presenting for Ophthalmic Examination in Baltimore, Maryland

**DOI:** 10.1167/tvst.12.11.7

**Published:** 2023-11-03

**Authors:** Alfred Vinnett, Zeshan Tariq, Jason A. Alvarez, Laura Andrews, Nneoma Okezie, Moran R. Levin, Mona A. Kaleem

**Affiliations:** 1 University of Maryland School of Medicine, Baltimore, MD, USA; 2 University of Pittsburgh School of Medicine, Pittsburgh, PA, USA; 3 Beach Eye Care, Virginia Beach, VA, USA; 4 The Eye Care Group, Waterbury, CT, USA; 5 Notre Dame of Maryland University, Baltimore, MD, USA; 6 Johns Hopkins University School of Medicine, Baltimore, MD, USA

**Keywords:** homelessness, vision-related quality of life (VRQOL), social determinants of health, baltimore, visual function index-14 (VF-14)

## Abstract

**Purpose:**

The coronavirus disease 2019 (COVID-19) pandemic is projected to drive 1.5 million Americans toward homelessness, adding to the 3.5 million currently affected. Homelessness poses both socioeconomic and public health challenges because housing status is a social determinant of health. Given ophthalmic health's importance in daily functioning, we characterized ophthalmic disease and vision-related quality of life (VRQOL) among a population experiencing homelessness in Baltimore, Maryland.

**Methods:**

Questionnaires, including a Visual Function Index-14 (VF-14) for measuring VRQOL, were administered among patients seeking eye examinations at Health Care for the Homeless (HCH) from October 2018 to March 2020.

**Results:**

One hundred sixty-two participants were enrolled in this study. The average age was 53 years. Participants’ most common vision concerns were blurry vision (70%) and desire for glasses (52%). Best corrected visual acuity (BCVA) measurements revealed significant vision loss (18%, *P* < 0.001). Physicians mostly diagnosed refractive error (77%), cataracts (36%), glaucoma/glaucoma suspect (25%), and dry eye (24%). Nearly half were referred to additional ophthalmic care (46%). VRQOL trends reflected functional vision categories (*P* = 0.042 and *P* = 0.021). The 1:1 VRQOL and BCVA comparison showed correlation (*rho* = −0.3, *P* < 0.001). Cronbach's alpha demonstrated VF-14 reliability (alpha = 0.92).

**Conclusions:**

We find high ophthalmic disease prevalence within a population experiencing homelessness. Comparison to studies worldwide reveals healthcare disparities despite healthcare system differences, suggesting a need for more targeted solutions. VF-14 is valid and reliable in assessing those experiencing homelessness. Intragroup VRQOL comparisons may reveal subgroup needs. It is imperative that future studies continue monitoring those experiencing homelessness.

**Translational Relevance:**

Validation of VF-14 will allow future studies to utilize this patient-oriented metric within populations experiencing homelessness.

## Introduction

Homelessness is a major social determinant of health affecting an estimated 3.5 million Americans annually.^1^ Individuals experiencing homelessness have mortality rates two to five times that of the age-matched general population^1^ and are more likely to exhibit comorbidities including cardiovascular disease,^2^ depression,^3^ and substance use disorders.^4^ Homelessness has a significant economic impact, as every individual chronically experiencing homelessness is expected to cost the US taxpayer $30,000 to $50,000 annually.^5^

In the years preceding the coronavirus disease 2019 (COVID-19) pandemic, the number of individuals experiencing homelessness has increased nationwide,^6^ and the COVID-19 pandemic is only expected to exacerbate these numbers.^7^ Projections estimate the pandemic may drive an additional 1.5 million Americans toward homelessness.^7^

A number of ophthalmic diseases have been identified in populations experiencing homelessness.^8^^–^^11^ Vision is of special importance due to its central role in maintaining quality of life and everyday functions.^12^ Individuals with counting fingers visual acuity reported a willingness to trade half of their remaining years for perfect vision.^12^ Furthermore, visual impairment can reduce the ability to work and can limit job opportunities.^13^^–^^16^ Understanding the impact of homelessness on ophthalmic diseases is especially important, considering the aging demographics of the US population^17^ including those experiencing homelessness.^18^ Older individuals are more likely to suffer from ophthalmic diseases,^19^ including dry eye,^20^ glaucoma,^21^ cataracts,^22^ diabetic retinopathy,^23^ and macular degeneration.^24^ Despite the importance of vision in performing activities of daily living, there are few studies that broadly examine both the ophthalmic diseases of Americans experiencing homelessness and the impact of homelessness on quality of life.

To address this gap in knowledge, we prospectively examined the prevalence of eye diseases and assessed vision-related quality of life (VRQOL) within a population experiencing homelessness that sought comprehensive ophthalmic examination at the Health Care for the Homeless (HCH) eye clinic in downtown Baltimore, Maryland.

## Methods

### Patient Recruitment

We conducted a prospective, cross-sectional survey study of patients presenting to the downtown Baltimore HCH eye clinic from October 2018 to March 2020. HCH is a member of the National Health Care for the Homeless Council and provides subsidized healthcare for individuals defined as homeless by the federal government's Health Resources and Services Administration. Each patient seen at HCH and its eye clinic met the definition of a “homeless individual” as defined under the Public Health Service Act.^25^ Individuals covered under the definition include those living in transitional housing or in facilities providing temporary living accommodations.^25^ This study was approved by the Human Research Protections Office/Institutional Review Board of University of Maryland, Baltimore. Data collection was initiated only after the study's approval, and the research was conducted in compliance with institutional policies and in adherence to the Declaration of Helsinki. Patients 18 years of age or older who could understand the survey in English or Spanish were eligible for enrollment. Verbal consent was obtained by study team members after an explanation of the study, and Spanish-speaking individuals were enrolled via a phone interpreter. Patients who did not agree to participate went on to complete their visit at the eye clinic and were not included in the data collection process.

### Surveys

Surveys were administered to participants, and Spanish-translated versions were provided when appropriate. The survey consisted of a three-page questionnaire that anonymously gathered self-identified demographic information, including age, race, ethnicity, medical history, and living conditions. The survey also included a Visual Function Index-14 (VF-14) questionnaire to assess VRQOL.^26^ The VF-14 questionnaire described 14 activities, and participants indicated the difficulty of each applicable activity using a 5-category Likert scale. Its options consisted of “none,” “having little difficulty carrying out the task,” “having moderate difficulty carrying out the task,” “having great deal of difficulty carrying out the task,” and “unable to carry out the task,” and these answers were later scored zero through four respectively. Alternatively, participants could indicate that the activity was not applicable. The scores corresponding to applicable questions were averaged, multiplied by 25, then subtracted from 100. Jenks Natural Breaks optimization^27^ was used to cluster the final scores into three classes. We categorized participants with the highest VRQOL scores as having “Good VRQOL” and participants with the lowest VRQOL scores as having “Poor VRQOL.”

As an incentive, a US $5 gift card was offered for survey completion. In cases where participants were unable to read or comprehend our survey, research team members assisted participants’ understanding by reading prompts aloud, answering questions, and recording responses as needed.

### Eye Examination

Patient examinations were conducted by resident ophthalmologists from either University of Maryland Medical Center or Sinai Hospital of Baltimore. Examinations included visual acuity assessment, pupil examination, intraocular pressure measurement, refraction, slit lamp examination, and dilated examination. Any patients requiring further evaluation were referred to local academic ophthalmic clinics. Diagnostic information was obtained from the ophthalmologist immediately following the participant's examination. All data were collected at the time of the visit in a de-identified manner.

### Statistical Analyses

For continuous data, we applied a Kolmogorov-Smirnov test to assess for normality. No compared groups had both data sets pass the normality test, so all continuous data were compared using a two-tailed Mann-Whitney U test. All categorical data were analyzed using Pearson's chi-squared tests. In comparisons with the National Health and Nutrition Examination Survey (NHANES), the number of adults surveyed was calculated by subtracting the number of participants aged 12 to 17 years from the total number of NHANES participants.^28^ Best Corrected Visual Acuity (BCVA) scores were categorized into NHANES-defined groupings, with Normal Vision, Any Vision Loss, Visual Impairment, and Blindness being defined as 20/30 or better, 20/40 or worse, 20/40 to better than 20/200, and 20/200 or worse BCVA in the better-seeing eye, respectively.^28^ In addition, monocular vision loss was defined as 20/40 or worse BCVA in either eye.^28^ The *P* values less than 0.05 were considered significant, and statistical significances of less than 0.05, 0.01, and 0.001 were denoted by *, **, and ***, respectively. Visual acuity categories of counting fingers, hand movement, light perception, and no light perception were converted to logMAR values corresponding to Snellen visual acuities of 20/1000, 20/2000, 20/4000, and 20/4000, respectively.^26^ Weighted logMAR (WMAR) values were calculated using the following relation: WMAR = (0.75 × logMAR acuity in the better-seeing eye) + (0.25 × logMAR acuity in worse-seeing eye).^29^^,^^30^ Spearman's *Rho* was calculated using GraphPad Prism 9 (GraphPad Software, Inc.). The reliability and internal consistency of the VF-14 questionnaire was assessed using Cronbach's alpha coefficient,^31^ based on the responses from participants finding all VF-14 questions applicable. We used bar graphs to plot categorical data and box-and-whisker graphs and violin graphs to plot continuous data.

## Results

**Figure 1.**
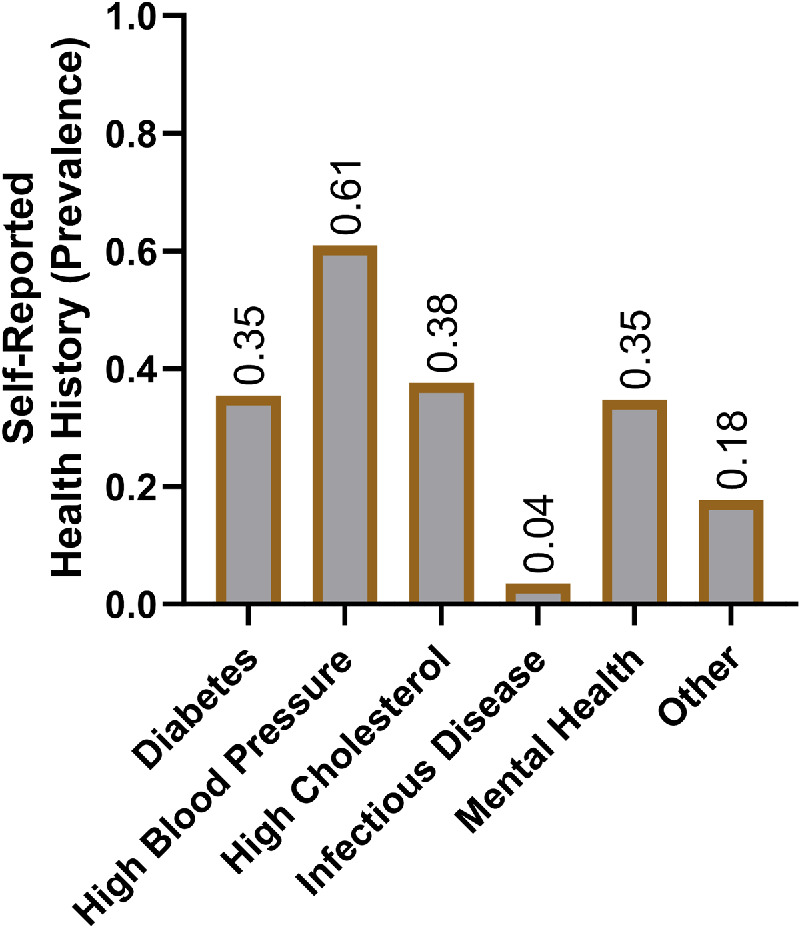
**Self-reported health history of surveyed participants.** Participants may select multiple options.

We surveyed 162 adults who sought ophthalmic care from the HCH Eye Clinic in downtown Baltimore, Maryland from October 2018 to March 2020. In total, we approached 184 individuals, with 18 declining participation and 4 falling under the age of 18 years. One of the 162 surveyed participants was excluded from analysis due to inability to confirm age. Self-identified demographic information may be found in Table 1. Our sample was equally composed of both self-identified men and self-identified women. Most participants identified as either Black/African American (50%) or Hispanic/Latino (39%) adults with a minority identifying as Asian (1%), American Indian/Alaska Native (1%), or White (12%) adults. A small subset of participants engaged in employment (15%). When asked in our general survey to select from a list of potential barriers to receiving ophthalmologic care, the most common selected answer was no insurance (46%). Other notable barriers included no transportation (17%) and inadequate insurance coverage (15%). Most participants relied upon public transportation (75%), whereas other patients reported walking to the clinic (22%) or relying upon family or friends for rides (16%). Self-reported medical history revealed that most individuals suffered from elevated blood pressure (61%), high cholesterol (38%), diabetes (35%), and mental health disease (35%; Fig. 1).

**Table 1.** Demographic and General Characteristics of Surveyed Participants

Characteristics	Prevalence	Participants (*n*)	Sample Size (*n*)
*Age, y*			
18-39	0.18	29	161
40-64	0.58	93	161
65-79	0.24	39	161
≥80	0.00	0	161
*Self-identified gender*			
Men	0.51	82	161
Women	0.49	79	161
*Self-identified race and ethnicity* ^*^			
Asian adults	0.01	1	161
Black or African American adults	0.50	80	161
Hispanic/Latino adults	0.39	62	161
American Indian or Alaska Native adults	0.01	1	161
White adults	0.12	20	161
*Work status*			
Working	0.15	24	159
Not working	0.85	135	159
*Eyecare barriers* ^*^			
Nothing	0.17	27	155
No insurance	0.46	72	155
Insurance does not cover	0.15	24	155
No transportation	0.17	26	155
No time	0.05	8	155
Other	0.15	24	155
*Transportation*			
Car	0.09	15	158
Ride from family or friends	0.16	26	158
Public transportation	0.75	118	158
Cab/taxi	0.08	13	158
Walk	0.22	34	158

* indicates that participants may select multiple options.

**Figure 2.**
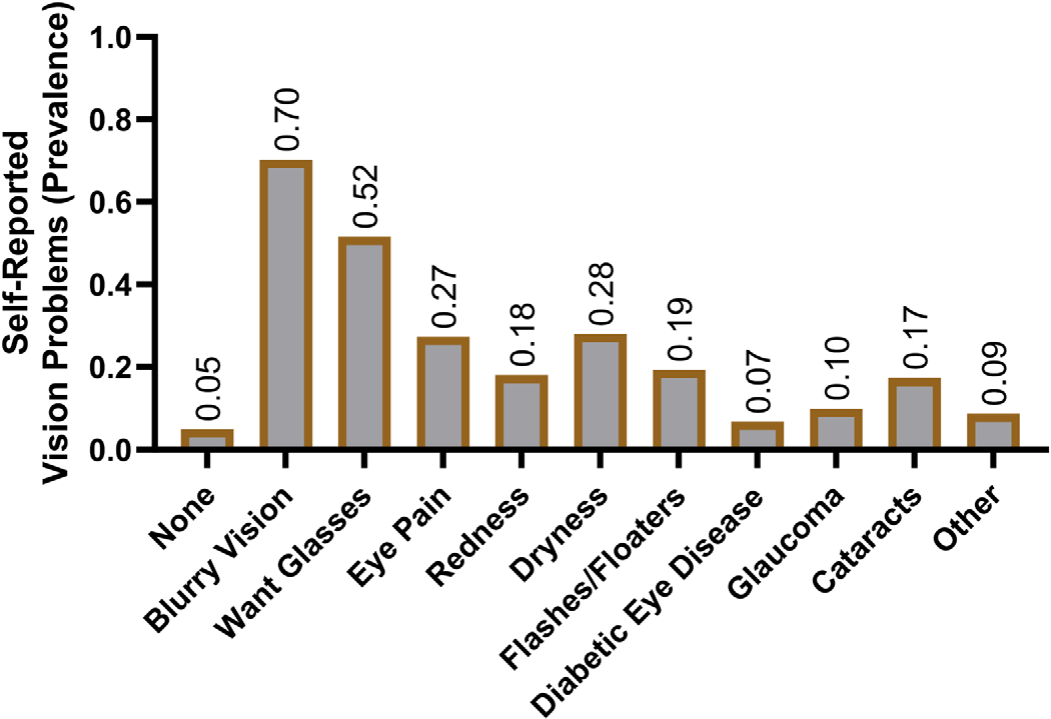
**Self-reported vision concerns of surveyed participants.** Participants may select multiple options.

**Figure 3.**
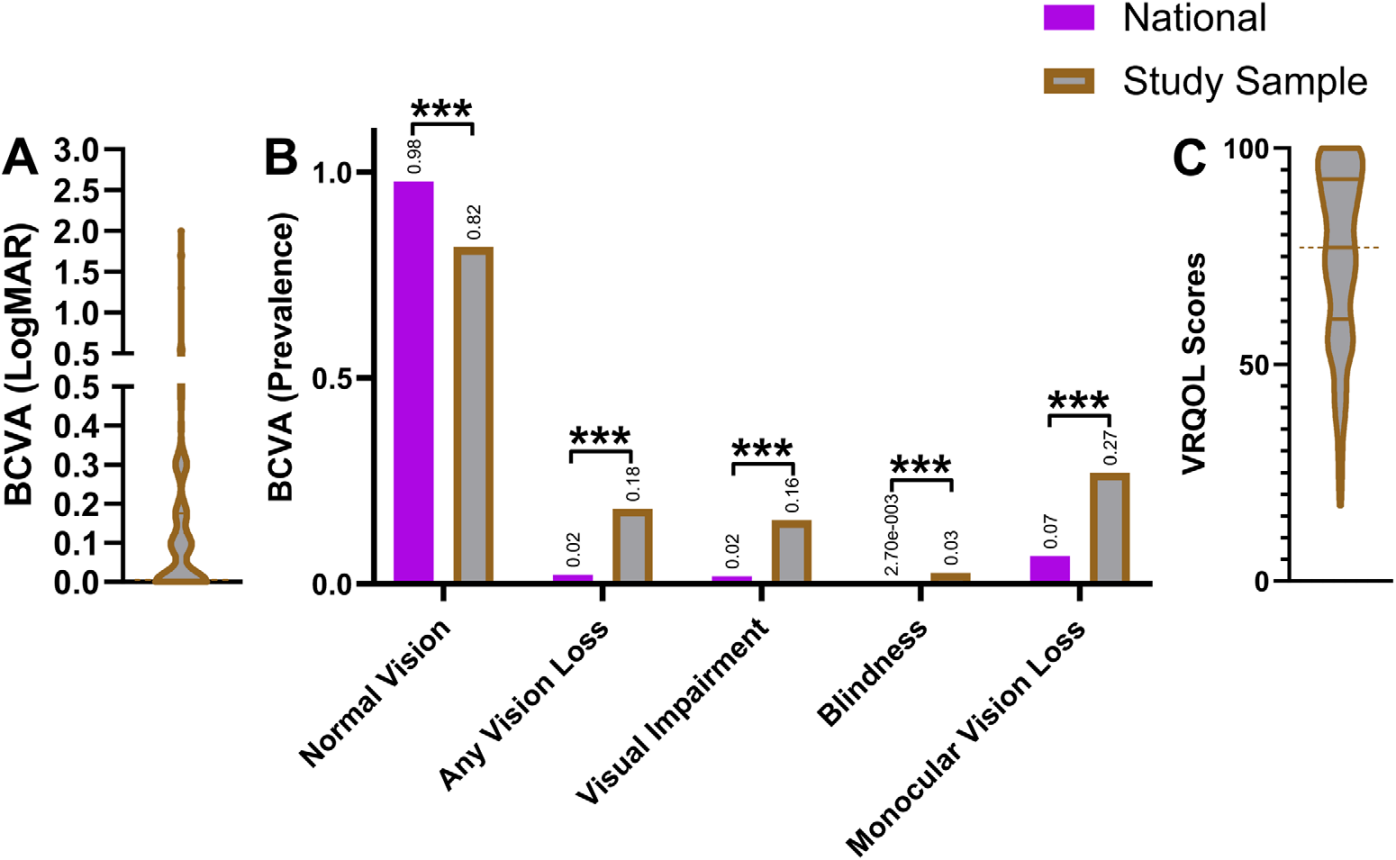
**BCVA and VRQOL of surveyed participants.** (**A**) BCVA of surveyed participants using better-seeing eye BCVA values. *Solid horizontal lines* represent the upper quartile, median, and lower quartile. The *dashed horizontal line* emphasizes the median. (**B**) BCVA comparison of randomized national sample versus surveyed participants. Normal Vision, Any Vision Loss, Visual Impairment, and Blindness are defined as 20/30 or better, 20/40 or worse, 20/40 to better than 20/200, and 20/200 or worse BCVA in the better-seeing eye, respectively.^28^ Monocular vision loss is defined as 20/40 or worse BCVA in either eye.^28^ Statistical significances of less than 0.001 are denoted by ***. (**C**) VRQOL scores of surveyed participants. *Solid lines* represent the upper quartile, median, and lower quartile. The *dashed line* emphasizes the median.

**Figure 4.**
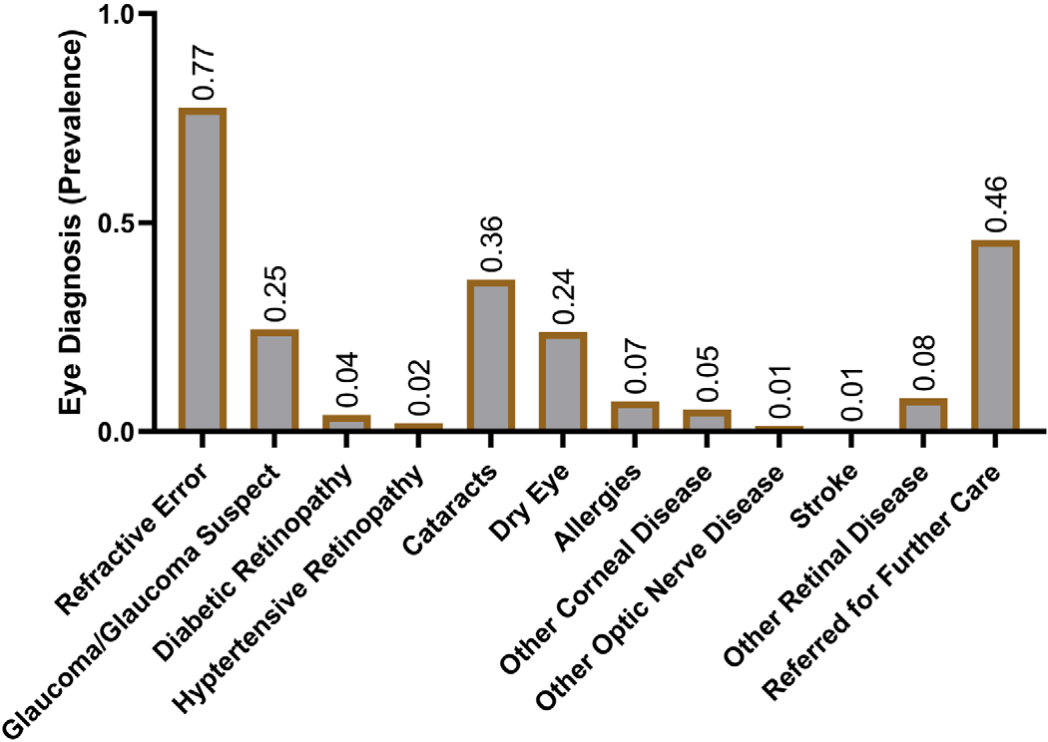
**Ophthalmologic diagnoses of surveyed participants at same day visit.** Participants may have multiple diagnoses.

The most common self-reported concerns were blurry vision (70%), seeking glasses (52%), dryness (28%), and eye pain (27%; Fig. 2). Conversely, some participants had no vision concerns (5%; see Fig. 2). The average logMAR score in the better-seeing eye was 0.14 (Fig. 3A), which fell between 20/25 and 20/30 on the Snellen visual acuity scale. Comparing against adults enrolled in NHANES^28^ revealed worse BCVA through decreased prevalence of normal vision (*P* < 0.001) and through increased prevalence of vision loss (*P* < 0.001), visual impairment (*P* < 0.001), blindness (*P* < 0.001), and monocular vision loss (*P* < 0.001; Fig. 3B). Most participants were diagnosed with refractive error (77%), followed by cataracts (36%), glaucoma/glaucoma suspect (25%), and dry eye (24%; Fig. 4). Forty-six percent of the participants were referred for further care at our hospital-based eye clinics (see Fig. 4).

Our study sample had an average VRQOL score of 75/100 (Fig. 3C), which corresponded to an average of “having little difficulty carrying out the task” across all applicable activities listed in the VF-14 questionnaire. Difficulty with activities that involved near vision were especially prominent (Table 2). The Cronbach's alpha coefficient for the VF-14 survey among participants finding all VF-14 questions applicable was 0.92 (*n* = 78), which indicated high internal consistency and reliability.

**Table 2.** Participant Responses to the VF-14 Questionnaire

Survey Question	No Difficulty	A Little Difficulty	Moderate Difficulty	Great Difficulty	Unable	N/A	Sample Size (*n*)
Q1: Reading small print	0.20	0.22	0.18	0.33	0.08	0.00	160
Q2: Reading book or newspaper	0.26	0.22	0.16	0.24	0.07	0.04	160
Q3: Reading large print book or newspaper	0.59	0.21	0.10	0.08	0.01	0.02	160
Q4: Recognizing people when close	0.64	0.21	0.04	0.09	0.01	0.01	160
Q5: Seeing steps, stairs, or curbs	0.59	0.22	0.09	0.07	0.01	0.03	160
Q6: Reading traffic, street, or store signs	0.49	0.25	0.15	0.08	0.00	0.03	160
Q7: Doing fine handwork	0.35	0.12	0.16	0.14	0.08	0.14	160
Q8: Writing checks or filling out forms	0.39	0.24	0.14	0.11	0.03	0.08	160
Q9: Playing card games, bingo, dominoes	0.46	0.14	0.12	0.08	0.01	0.19	160
Q10: Taking part in sports (bowling, handball, tennis, golf)	0.48	0.10	0.09	0.03	0.04	0.27	160
Q11: Cooking	0.61	0.19	0.11	0.04	0.00	0.06	160
Q12: Watching television	0.40	0.27	0.17	0.09	0.02	0.05	160
Q13: Driving during day	0.38	0.11	0.08	0.03	0.04	0.38	160
Q14: Driving at night	0.27	0.11	0.12	0.08	0.05	0.38	160

**Figure 5.**
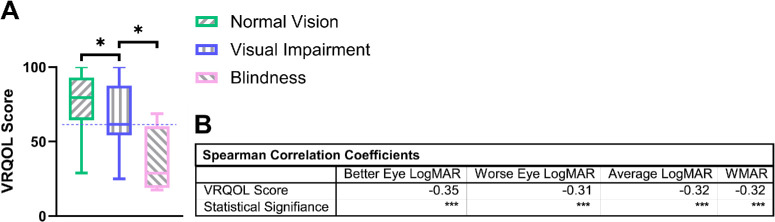
**BCVA versus VRQOL of surveyed participants.** (**A**) VRQOL score comparison of Normal Vision (20/30 or better) versus Visual Impairment (20/40 to better than 20/200) versus Blindness (20/200 or worse) in the better-seeing eye. Solid horizontal lines represent their respective color's upper quartile, median, and lower quartile. The *dashed horizontal line* emphasizes the median of the Visual Impairment group. Statistical significances of less than 0.05 are denoted by *. (**B**) VRQOL correlations with BCVA scores. Statistical significances of less than 0.001 are denoted by ***.

To validate the VF-14 measure of VRQOL for those experiencing homelessness, we first separated our sample into the predefined BCVA cutoffs defined by NHANES and compared VRQOL between these predefined groups. Specifically, NHANES defined Normal Vision as 20/30 or better in the better-seeing eye, Visual Impairment as 20/40 to better than 20/200 in the better-seeing eye, and Blindness as 20/200 or worse in the better-seeing eye.^28^ Comparison of all classes showed a statistically significant difference in VRQOL in a step-down manner with Normal Vision being significantly higher than Visual Impairment (*P* = 0.042) and Blindness being significantly lower than Visual Impairment (*P* = 0.021; Fig. 5A). This was further corroborated through a one-to-one correlation of BCVA against VRQOL scores, with comparisons of VRQOL scores against the better-seeing eye BCVA, worse-seeing eye BCVA, average eye BCVA, and WMAR having Spearman correlation coefficients of −0.35, −0.31, −0.32, and −0.32, respectively (Fig. 5B). All four comparisons were statistically significant (*P* < 0.001; see Fig. 5B).

**Figure 6.**
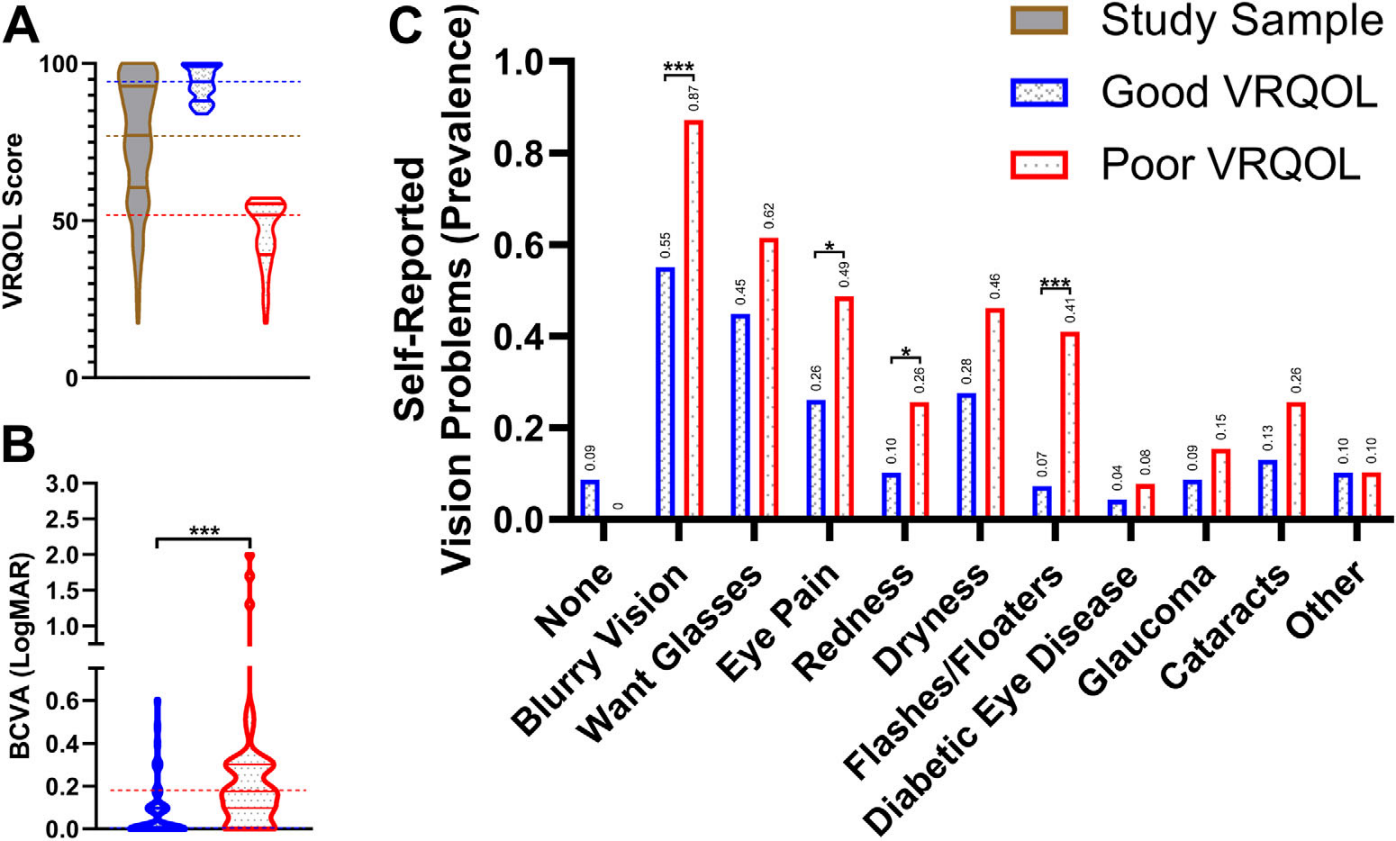
**“Good VRQOL” versus “Poor VRQOL” scores.** (**A**) “Good VRQOL” and “Poor VRQOL” Jenks clusters. *Solid horizontal lines* represent their respective color's upper quartile, median, and lower quartile. *Dashed horizontal lines* emphasize their respective color's median. (**B**) Better-seeing eye BCVA comparison of “Good VRQOL” versus “Poor VRQOL” scores. *Solid horizontal lines* represent their respective color's upper quartile, median, and lower quartile. *Dashed horizontal lines* emphasize their respective color's median. Statistical significance of less than 0.001 is denoted by ***. (**C**) Self-reported vision problem comparison of “Good VRQOL” versus “Poor VRQOL” scores. Participants may select multiple vision problem options. Statistical significances of less than 0.05 and 0.001 are denoted by * and ***, respectively.

**Figure 7.**
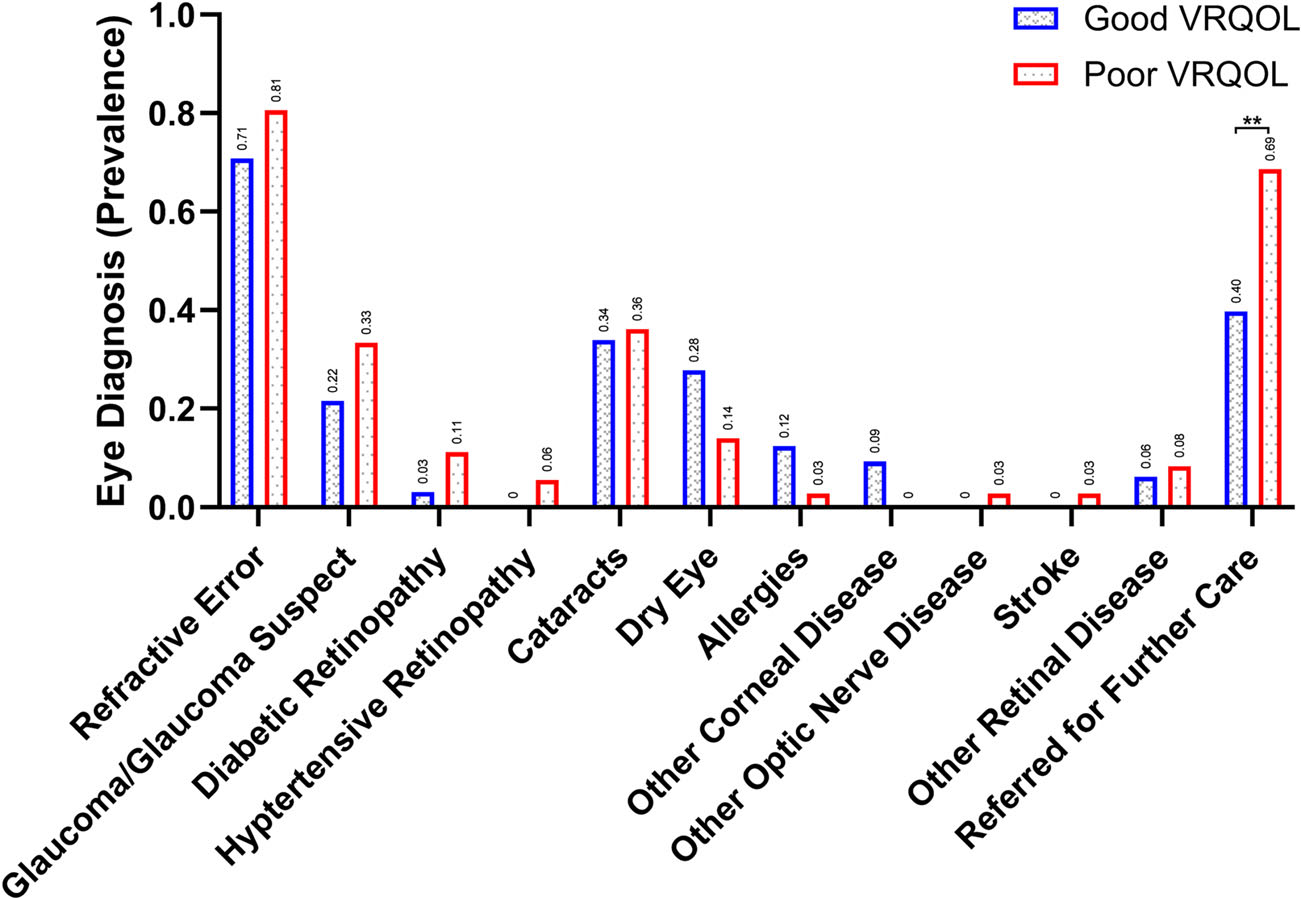
**Ophthalmologic diagnoses comparison of “Good VRQOL” versus “Poor VRQOL” scores.** Participants may have multiple diagnoses. Statistical significance of less than 0.01 is denoted by **.

Next, we correlated VRQOL groupings with both self-reported vision problems and disease diagnoses. Violin plotting of our sample's VRQOL scores showed three distinct clusters (see Fig. 3C), so we applied the Jenks clustering algorithm to separate our data into three classes based on VRQOL scores (Fig. 6A). We used the Jenks clustering results to define “Good VRQOL” scores as those with values of 84 through 100 and “Poor VRQOL” scores as those with values of 18 through 57. Comparison of the “Good VRQOL” and “Poor VRQOL” groups demonstrated better median BCVA in the “Good VRQOL” group (20/20) relative to the “Poor VRQOL” group (20/30, *P* < 0.001; Fig. 6B) and highlighted higher prevalence of several self-reported vision problems among the “Poor VRQOL” group relative to the “Good VRQOL” group (Fig. 6C). Significant differences between “Good VRQOL” and “Poor VRQOL” were observed when comparing blurry vision (55% vs. 87%, *P* < 0.001), eye pain (26% vs. 49%, *P* = 0.017), redness (10% vs. 26%, *P* = 0.034), and flashes/floaters (7% vs. 41%, *P* < 0.001) categories (see Fig. 6C). Despite these statistically significant differences in self-reported vision problems, we did not find statistically significant differences in prevalence of diagnosed eye disease between “Good VRQOL” and “Poor VRQOL” groups. However, there was a large increase in referral rates for further subspecialty ophthalmic care in the “Poor VRQOL” group (69%) relative to the “Good VRQOL” group (40%, *P* = 0.006; Fig. 7).

Of note, sample sizes for each figure, including subsample sizes where applicable, may be found in the Supplemental Tables S1 through S7. These tables are numbered sequentially in the order of the figures they correspond to.

## Discussion

In this study, we surveyed participants experiencing homelessness who sought eyecare at HCH in downtown Baltimore, Maryland, in order to identify features and rates of ophthalmic disease and to validate the VF-14 questionnaire in this population. We found reduced visual acuity within our sample compared to adults enrolled in NHANES,^28^ and our most common diagnoses were refractive error, glaucoma/glaucoma suspect, cataracts, and dry eye. We validated the VF-14 questionnaire for those experiencing homelessness by demonstrating expected trends in BCVA between NHANES-defined BCVA categories and by demonstrating consistency of 1:1 BCVA to VRQOL correlations with those of other VF-14 validity studies.

Surveying participants who sought eyecare at HCH revealed significant needs. Most participants sought care due to blurry vision (70%) or desire for glasses (52%). Correspondingly, 77% of participants were diagnosed with refractive error, and the percentage of refractive error in our study exceeded that of other similar studies (Table 3).^8^^,^^9^^,^^11^^,^^32^^–^^36^ However, regardless of refractive error, our study population would still have overall poor vision, as the BCVA values of our study population were worse than those of NHANES (see Fig. 3B). Subsequently, our sample had a heightened prevalence of ophthalmic disease that even exceeded those of similar studies examining eye disease in those experiencing homelessness (see Table 3),^8^^,^^9^^,^^11^^,^^32^^–^^36^ with the exception of diabetic retinopathy in one of those studies.^34^

**Table 3.** Eye Diagnosis Comparison Between Studies Examining Populations Experiencing Homelessness

First Author	Current Study	Jiang^8^	Pitz^11^	Noel^9^	Ho^33^	Sawers^36^	Henstenburg^32^	Nnamani Silva^34^	Quigley^35^
City, country	Baltimore, MD, USA	Toronto, Canada	Mainz and Frankfurt, Germany	Toronto, Canada	Los Angeles, CA, USA	London, UK	Philadelphia, PA, USA	San Francisco, CA, USA	Baltimore, MD, USA
Year published	2023	2020	2005	2015	1997	2017	2019	2020	2002
Average age	53	–	49	–	30	49	–	51	50
Median age	55	53	–	48	–	–	–	–	48
Refractive error %	0.77	0.48	–	–	0.54	0.43	0.45	0.70	0.53
Glaucoma/glaucoma suspect %	0.25	0.10	–	0.06	0.02	0.08	0.05	0.20	0.13
Cataracts %	0.36	0.10	0.08	0.04	0.05	0.19	0.29	0.20	0.15
Diabetic retinopathy %	0.04	–	0.02	–	–	–	0.03	0.11	–
Dry eye %	0.24	0.01	–	–	–	–	–	0.08	–
Hypertensive retinopathy %	0.02	–	–	–	–	–	0.01	0.01	–
Referral rates	0.46	0.43	–	–	–	0.10	–	–	–

Despite expansion of Medicaid within Maryland via the Affordable Care Act, there remains a present healthcare need for interventions to improve vision and eye health in those experiencing homelessness. Participants identified insurance-related barriers (i.e. lack of insurance [46%] and/or lack of coverage [15%]) as the most likely to affect healthcare access, which is greater than the 8% of people nationally without health insurance coverage.^37^ Besides expansion of insurance coverage, solutions to this issue may additionally involve greater resource allocation toward addressing the most prevalent eyecare needs through resource-efficient means. Particularly, refractive error needs were noted to be the highest among vision-related needs in our study, and this finding has been consistent across major cities within the United States and in countries with universal public healthcare (see Table 3).^8^^,^^32^^–^^36^ Given that our study reported the highest prevalence of refractive error needs (77%) relative to prior studies (see Table 3),^8^^,^^32^^–^^36^ the true prevalence of refractive error needs in those experiencing homelessness may have been underappreciated. Additionally, improving vision through addressing uncorrected refractive error is a relatively resource-efficient intervention. Uncorrected refractive error can be addressed through corrective lenses, which are lower-cost treatments than treatments for other eye conditions like cataracts, glaucoma, and age-related macular degeneration.^38^ Therefore, accessible refractive error measurement and distribution of spectacles should be increasingly prioritized as relatively cost-effective interventions in improving the vision of populations who are experiencing homelessness and experiencing insurance-related barriers.

Our study validated the VF-14 questionnaire as a reliable measure of VRQOL in those experiencing homelessness. Although correlating VRQOL and visual acuity scores in a population experiencing homelessness was not novel,^39^ attempting to validate this measure in a population experiencing homelessness was. We selected the VF-14 questionnaire over other established and widely used VRQOL-measuring tools because this 14-question survey is relatively short, has been widely validated, and has internal consistency.^40^ Despite being initially developed for patients with cataract,^26^ the VF-14 survey has demonstrated versatility, as it has successfully assessed VRQOL for patients with age-related macular degeneration, diabetic retinopathy, glaucoma, corneal graft, retinal detachment, low vision, and other conditions.^40^ In addition, the VF-14 questionnaire is known to have internal consistency.^26^^,^^41^^,^^42^ In fact, our VF-14 results had a Cronbach's alpha of 0.92, which corresponded to a high level of reliability that was stronger than those reported in similar studies.^26^^,^^41^^,^^42^

To validate the VF-14 questionnaire for those experiencing homelessness, we first separated our sample into the predefined BCVA cutoffs defined by NHANES and compared VRQOL between these predefined groups (see Fig. 5A). We specifically chose the predefined BCVA cutoffs of NHANES over those used in other VF-14 validation studies^26^^,^^41^^,^^42^ to improve external validity, as NHANES is the government program recognized for being the basis of national health measurements in the United States.^43^ Comparison of the clusters revealed a reduction in VRQOL scores across all three classes (see Fig. 5A). Additionally, one to one analysis found correlations when comparing VRQOL scores to better-seeing eye BCVA (*rho* = −0.35), worse-seeing eye BCVA (*rho* = −0.31), average eye BCVA (*rho* = −0.32), and WMAR (*rho* = −0.32; see Fig. 5B). All four Spearman correlation coefficients were stronger than those reported by Steinberg et al. (1994),^26^ although only the Spearman correlation coefficient associated with the better-seeing eye was stronger than its counterpart from Linder et al. (1999).^41^ Overall, our findings demonstrate consistency with prior literature regarding the correlation of various BCVA classifications and VRQOL scores.

Violin plotting of our sample's VRQOL scores demonstrated three distinct clusters (see Fig. 3C). To investigate characteristics of these natural distributions, we delineated groups from these clusters (see Fig. 6A) using Jenks Natural Breaks optimization, as this method objectively categorizes data in a way that minimizes intragroup variance. To better understand the needs of those with the lowest VRQOL scores, we focused our investigation on this group, which we defined as “Poor VRQOL.” We compared “Poor VRQOL” to the group with the highest VRQOL, which we defined as “Good VRQOL” (see Fig. 6A). Not only were there expected differences in BCVA (see Fig. 6B), but also there was an increase in self-reported vison concerns within the “Poor VRQOL” group (see Fig. 6C). These findings included blurry vision, eye pain, eye redness, and flashes/floaters among the “Poor VRQOL” group relative to the “Good VRQOL” group (see Fig. 6C). Interestingly, we did not observe an increase in any specific disease category among the “Poor VRQOL” group but did find a large increase in ophthalmic referrals (40% vs. 69%, *P* = 0.006). This finding suggests that the “Poor VRQOL” group has more complex pathology, requiring greater medical attention. Overall, we find VF-14 to be a valid and reliable measure of VRQOL within a population experiencing homelessness, and it may serve as a tool for future studies examining homelessness.

Of note, the utility of the “Good VRQOL” and “Poor VRQOL” comparisons may be limited. The VF-14 has not been specifically validated in several “Self-Reported Vision Problem” and “Eye Diagnosis” categories, including those showing significance between “Good VRQOL” and “Poor VRQOL” groups. However, the VF-14 is a flexible tool that has been validated for a variety of ophthalmic conditions,^40^ with some sharing features that were significant between “Good VRQOL” and “Poor VRQOL.” Thus, the impact of absent VF-14 validation in these categories may be somewhat mitigated.

Another limitation of our study was that the relatively advanced age of our sample may have contributed to the high prevalences of eye morbidity relative to NHANES and to the prior referenced studies (see Tables 1 and 3).^8^^,^^9^^,^^11^^,^^28^^,^^32^^–^^36^ However, demographic aging of the population experiencing homelessness is an emerging characteristic.^18^^,^^44^^–^^46^ Thus, advanced age within our sample and other recent studies examining homelessness may be a more limited confounder of eye morbidity, especially for age-related conditions like dry eye, cataract, and macular disease (see Fig. 4).

In addition, our study was limited by non-random sampling of participants. We only surveyed those who presented for examination, allowing for selection bias where only those with an ophthalmic concern were referred for an examination. A possible solution would be to randomly select individuals experiencing homelessness and residing in a shelter, as previously reported.^47^ However, not all individuals experiencing homelessness live in such an accommodation. Instead, it may be better to attempt to mitigate selection bias by comparing the sample of participants experiencing homelessness and presenting for ophthalmic examination to a demographically comparable but housed population presenting for ophthalmic examination. This solution would allow for a more balanced comparison between those experiencing and not experiencing homelessness, while maintaining a similar sampling method to the current study.

Finally, the self-reported nature of the medical history may have introduced reporting bias, potentially affecting the accuracy of results (see Fig. 1). Future studies may choose to incorporate a retrospective chart review to augment or replace self-reported data to increase the accuracy of findings.

We find a population experiencing homelessness with unaddressed ophthalmic needs, and we expect exacerbation of these needs given both demographic aging and recent expansion of homelessness from the COVID-19 pandemic. It is imperative that we continue monitoring populations experiencing homelessness as they change in order to anticipate and better address their needs. Additionally, we hope that validation of VF-14 will allow for a more patient-based approach when studying eye health of those experiencing homelessness.

## Supplementary Material

Supplement 1
