# Supplementary material for: Eye Disease Prevalence and VF-14 Validation Among Patients Experiencing Homelessness and Presenting for Ophthalmic Examination in Baltimore, Maryland
Source: Transl Vis Sci Technol. 2023 Nov 3;12(11):7. doi: 10.1167/tvst.12.11.7 (PMC10629521; doi:10.1167/tvst.12.11.7)
Supplement: Supplement 1 [file tvst-12-11-7_s001.pdf]

Table S1. Prevalence, Participants, and Sample Sizes of Self-Reported Health History of Surveyed Participants

**Characteristics**	**Prevalence**	**Participants (n)**	**Sample Size (n)**
Diabetes	0.35	50	141
High Blood Pressure	0.61	86	141
High Cholesterol	0.38	53	141
Infectious Disease	0.04	5	141
Mental Health	0.35	49	141
Other	0.18	25	141

Participants may select multiple options.

Table S2. Prevalence, Participants, and Sample Sizes of Self-Reported Vision Concerns of Surveyed Participants

Characteristics	Prevalence	Participants (n)	Sample Size (n)
None	0.05	8	161
Blurry Vision	0.70	113	161
Want Glasses	0.52	83	161
Eye Pain	0.27	44	161
Redness	0.18	29	161
Dryness	0.28	45	161
Flashes/Floaters	0.19	31	161
Diabetic Eye Disease	0.07	11	161
Glaucoma	0.10	16	161
Cataracts	0.17	28	161
Other	0.09	14	161

Participants may select multiple options.

Table S3. Prevalence, Participants, and Sample Sizes of BCVA and VRQOL of Surveyed Participants

Characteristics	Prevalence	Participants (n)	Sample Size (n)
BCVA of Surveyed Study Sample			
Study Sample	N/A	N/A	148
BCVA Comparison of Randomized National Sample vs Surveyed Study Sample			
Normal Vision (National)	0.98	23915	24470
Normal Vision (Study Sample)	0.82	121	148
Any Vision Loss (National)	0.02	555	24470
Any Vision Loss (Study Sample)	0.18	27	148
Visual Impairment (National)	0.02	458	24470
Visual Impairment (Study Sample)	0.16	23	148
Blindness (National)	0.00	66	24470
Blindness (Study Sample)	0.03	4	148
Monocular Vision Loss (National)	0.07	1835	27200
Monocular Vision Loss (Study Sample)	0.27	40	148
VRQOL Scores			
Study Sample	N/A	N/A	160

Normal Vision, Any Vision Loss, Visual Impairment, and Blindness are defined as 20/30 or better, 20/40 or worse, 20/40 to better than 20/200, and 20/200 or worse BCVA in the better-seeing eye respectively.[28] Monocular vision loss is defined as 20/40 or worse BCVA in either eye.[28]

Table S4. Prevalence, Participants, and Sample Sizes of Ophthalmologic Diagnoses of Surveyed Participants at Same Day Visit

**Characteristics**	**Prevalence**	**Participants (n)**	**Sample Size (n)**
Refractive Error	0.77	117	151
Glaucoma/Glaucoma Suspect	0.25	37	151
Diabetic Retinopathy	0.04	6	151
Hypertensive Retinopathy	0.02	3	151
Cataracts	0.36	55	151
Dry Eye	0.24	36	151
Allergies	0.07	11	151
Other Corneal Disease	0.05	8	151
Other Optic Nerve Disease	0.01	2	151
Stroke	0.01	1	151
Other Retinal Disease	0.08	12	151
Referred for Further Care	0.46	68	148

Participants may have multiple diagnoses.

Table S5. Prevalence, Participants, and Sample Sizes of BCVA vs VRQOL of Surveyed Participants

Characteristics	Prevalence	Participants (n)	Sample Size (n)
BCVA Groups vs VRQOL Scores			
Normal Vision	0.82	120	147
Visual Impairment	0.16	23	147
Blindness	0.03	4	147
VRQOL Correlations with BCVA Scores			
Better Eye BCVA	N/A	N/A	147
Worse Eye BCVA	N/A	N/A	147
Average BCVA	N/A	N/A	147
WMAR	N/A	N/A	147

Table S6. Prevalence, Participants, and Sample Sizes of Good vs Poor VRQOL Scores

Characteristics	Prevalence	Participants (n)	Sample Size (n)
Good vs Poor VRQOL Scores			
Study Sample	N/A	N/A	160
Good VRQOL	0.43	69	160
Poor VRQOL	0.24	39	160
BCVA Comparison of Good vs Poor VRQOL Scores			
Good VRQOL	0.44	64	147
Poor VRQOL	0.24	36	147
Self-Reported Vision Problems Comparison of Good vs Poor VRQOL Scores			
None (Good VRQOL)	0.09	6	69
None (Poor VRQOL)	0.00	0	39
Blurry Vision (Good VRQOL)	0.55	38	69
Blurry Vision (Poor VRQOL)	0.87	34	39
Want Glasses (Good VRQOL)	0.45	31	69
Want Glasses (Poor VRQOL)	0.62	24	39
Eye Pain (Good VRQOL)	0.26	18	69
Eye Pain (Poor VRQOL)	0.49	19	39
Redness (Good VRQOL)	0.10	7	69
Redness (Poor VRQOL)	0.26	10	39
Dryness (Good VRQOL)	0.28	19	69
Dryness (Poor VRQOL)	0.46	18	39
Flashes/Floaters (Good VRQOL)	0.07	5	69
Flashes/Floaters (Poor VRQOL)	0.41	16	39
Diabetic Eye Disease (Good VRQOL)	0.04	3	69
Diabetic Eye Disease (Poor VRQOL)	0.08	3	39
Glaucoma (Good VRQOL)	0.09	6	69
Glaucoma (Poor VRQOL)	0.15	6	39
Cataracts (Good VRQOL)	0.13	9	69
Cataracts (Poor VRQOL)	0.26	10	39
Other (Good VRQOL)	0.10	7	69
Other (Poor VRQOL)	0.10	4	39

Participants may select multiple vision problem options.

Table S7. Prevalence, Participants, and Sample Sizes of Ophthalmologic Diagnoses

Comparison of Good vs Poor VRQOL Scores

Characteristics	Prevalence	Participants (n)	Sample Size (n)
Refractive Error (Good VRQOL)	0.71	46	65
Refractive Error (Poor VRQOL)	0.81	29	36
Glaucoma/Glaucoma Suspect (Good VRQOL)	0.22	14	65
Glaucoma/Glaucoma Suspect (Poor VRQOL)	0.33	12	36
Diabetic Retinopathy (Good VRQOL)	0.03	2	65
Diabetic Retinopathy (Poor VRQOL)	0.11	4	36
Hypertensive Retinopathy (Good VRQOL)	0.00	0	65
Hypertensive Retinopathy (Poor VRQOL)	0.06	2	36
Cataracts (Good VRQOL)	0.34	22	65
Cataracts (Poor VRQOL)	0.36	13	36
Dry Eye (Good VRQOL)	0.28	18	65
Dry Eye (Poor VRQOL)	0.14	5	36
Allergies (Good VRQOL)	0.12	8	65
Allergies (Poor VRQOL)	0.03	1	36
Other Corneal Disease (Good VRQOL)	0.09	6	65
Other Corneal Disease (Poor VRQOL)	0.00	0	36
Other Optic Nerve Disease (Good VRQOL)	0.00	0	65
Other Optic Nerve Disease (Poor VRQOL)	0.03	1	36
Stroke (Good VRQOL)	0.00	0	65
Stroke (Poor VRQOL)	0.03	1	36
Other Retinal Disease (Good VRQOL)	0.06	4	65
Other Retinal Disease (Poor VRQOL)	0.08	3	36
Referred for Further Care (Good VRQOL)	0.40	25	63
Referred for Further Care (Poor VRQOL)	0.69	24	35

Participants may have multiple diagnoses.
